# An Autologous Muscle Tissue Expansion Approach for the Treatment of Volumetric Muscle Loss

**DOI:** 10.1089/biores.2015.0009

**Published:** 2015-03-01

**Authors:** Catherine L. Ward, Lisa Ji, Benjamin T. Corona

**Affiliations:** US Army Institute of Surgical Research, Extremity Trauma and Regenerative Medicine, Fort Sam Houston, Texas.

**Keywords:** physiology, tissue engineering, skeletal muscle, injury

## Abstract

Volumetric muscle loss (VML) is a hallmark of orthopedic trauma with no current standard of care. As a potential therapy for some VML indications, autologous minced muscle grafts (1 mm^3^ pieces of muscle) are effective in promoting remarkable *de novo* fiber regeneration. But they require ample donor muscle tissue and therefore may be limited in their application for large clinical VML. Here, we tested the hypothesis that autologous minced grafts may be volume expanded in a collagen hydrogel, allowing for the use of lesser autologous muscle while maintaining regenerative and functional efficacy. The results of the study indicate that 50% (but not 75%) less minced graft tissue suspended in a collagen hydrogel promoted a functional improvement similar to that of a 100% minced graft repair. However, approximately half of the number of fibers regenerated *de novo* with 50% graft repair. Moreover, the fibers that regenerated had a smaller cross-sectional area. These findings support the concept of using autologous minced grafts for the regeneration of muscle tissue after VML, but indicate the need to identify optimal carrier materials for expansion.

## Introduction

Muscle weakness and sub-optimal limb function are commonly a part of the sequelae of orthopedic trauma, even after successful limb salvage.^1,2^ The volumetric loss of skeletal muscle (volumetric muscle loss [VML]) often underlies persistent functional deficits after trauma, and there is no standard of care for the regeneration of the lost muscle tissue. Autologous free or rotational muscle grafts are commonly used for soft tissue replacement after severe open fracture to support bone regeneration, but not for the purpose of restoring muscle function.^3^ In some instances, functional whole skeletal muscle grafts have been successfully transplanted.^4^ And a breadth of animal studies have indicated that whole skeletal muscle grafts with surgically restored tendon attachment and neurovascular supply may be sufficiently large for clinical relevance.^5^ However, the availability of an adequate size donor muscle and potential donor site morbidity may limit the treatment of large VML injuries using this approach.

As a guide, the gold standard treatment for segmental bone defects involves transplantation of autologous bone grafts.^6^ However, in recognition of the limited availability of autologous bone for grafting, biomaterials are effective as conductive agents. For example, co-delivery of demineralized bone matrices (e.g., bone allograft) with autologous bone grafts is an effective means to volumetrically expand bone graft-mediated regeneration.^7,8^ Although differences exist in the mechanisms underlying bone and muscle regeneration, an analogous approach using autologous muscle grafts is reasonable for the repair of VML. As an alternative to *replacing* muscle tissue with whole muscle grafts, muscle *regeneration* may be achieved using autologous minced muscle grafts. Whole muscles minced into small pieces (1 mm^3^) maintain the capability to regenerate a functional muscle, although the regenerated fibers may be misaligned.^9–12^ More recently, minced muscle grafts were effective in promoting *de novo* muscle fiber regeneration and functional recovery in a rodent VML model,^13^ and minced grafts were successful in improving urinary incontinence in humans.^14^ An advantage of autologous minced versus whole muscle grafts is the potential for volume expansion in a myoconductive biomaterial, that is, the potential to regenerate a relatively large volume of muscle with a reduced amount of donor tissue.

The purpose of this study was to investigate the expansion capacity of autologous minced muscle grafts for the treatment of VML. This initial investigation involved the transplantation of a titrated amount of autologous minced skeletal muscle grafts delivered in a standard collagen hydrogel to the site of injury, the tibialis anterior (TA) muscle, in a rat VML model. Collagen hydrogel was chosen as an initial expansion biomaterial, because collagen I is the primary constituent of skeletal muscle extracellular matrix (ECM) and collagen-based gels and ECMs are available for clinical use.^15,16^ 100% minced graft and nonrepaired muscles served as positive and negative treatment controls, respectively, to establish upper- and lower-bound limits of regeneration in this VML model.

## Materials and Methods

### Experimental design

Male Lewis rats were divided among five experimental groups that each had VML injury in the left TA muscle: (1) no repair (negative treatment control), (2) repair with collagen hydrogel (0% minced graft), and repair with a (3) 25%, (4) 50%, or (5) 100% (positive treatment control) defect volume replacement of autologous minced grafts (e.g., for a 100 mg tissue defect, 100 mg of minced graft was replaced in the 100% group). The 25% and 50% volume graft replacement was suspended in collagen hydrogel to determine their capacity for expansion. Only for the 100% volume minced graft, the replacement group was a subset of rats (*n*=3) repaired with syngeneic TA muscle grafts from GFP-Lewis rats. Rats survived to 8 weeks postinjury, at which time the injured and contralateral uninjured TA muscle's strength was assessed *in vivo* and the muscles were harvested for histological and morphological analysis.

### Animals

This work has been conducted in compliance with the Animal Welfare Act, the implementation of Animal Welfare Regulations and in accordance with the principles of the Guide for the Care and Use of Laboratory Animals. All animal procedures were approved by the United States Army Institute of Surgical Research Institutional Animal Care and Use Committee. Adult male Lewis rats (Harlan Laboratories) and GFP-Lewis rats (SD-Tg(GFP)Bal), Rat Resource and Research Center (RRRC), University of Missouri, Columbia, MO were housed in a vivarium accredited by the Association for Assessment and Accreditation of Laboratory Animal Care International, and provided with food and water ad libitum. A total of 34 rats were used in this study.

### TA muscle VML injury

VML was surgically created in similar fashion to that previously reported.^2,13,17^ The TA and underlying extensor digitorum longus (EDL) muscles were separated using blunt dissection. A punch biopsy (6 mm) was performed through the middle third of the TA muscle, and the biopsied tissue was removed (∼75 mg). Sustained release buprenorphine (72 h) was delivered (1.2 mg/kg SC) before surgery for pain.

### Construct preparation

Autologous minced grafts were derived from the original TA muscle tissue that was excised to make the VML defect. The excised muscle tissue was minced into ∼1 mm^3^ pieces. The minced grafts were then suspended in a collagen hydrogel; rat tail collagen I (Becton Dickinson) was diluted in 2.5× Dulbecco's modified Eagle's medium (Gibco) to create a 3 mg/mL solution and kept on ice while the minced graft was prepared. After mincing, the desired volume (0–50%) of muscle tissue was added to a well of a 48-well culture plate. A volume of collagen hydrogel was added to the well to bring the final volume to 400 μL (i.e., the volume of muscle tissue was calculated based on muscle density), which filled the defect area. The mixture was stirred to create a homogenous distribution of minced grafts within the construct. The collagen constructs were then allowed to crosslink at 37°C for ∼45 min, after which time the constructs were transplanted to the VML defect. Fascia and skin were closed by suturing and stapling each layer, respectively.

### In vivo functional assessment

Isolated TA muscle *in vivo* functional properties were measured in anesthetized rats (isoflurane 1.5–2.0%) in both legs as previously described.^13^ A nerve cuff with multistranded stainless steel (632; Cooner Wire) wire electrodes was implanted in each leg around the peroneal nerve. Legs were tested separately and in randomized order. The foot was strapped using silk surgical tape to a footplate attached to a dual-mode muscle lever system (Mod. 305b; Aurora Scientific, Inc.). The knee was secured on either side using a custom-made mounting system, and the knee and ankle were positioned at right angles. Optimal voltage (2–5 V) was set with a series of tetanic contractions (5–10 contractions; 150 Hz, 0.1 msec pulse width, 400 msec train). Then, a skin incision was made at the antero-lateral aspect of the ankle and the distal EDL muscle tendon and extensor hallicus longus muscle was isolated and severed above the retinaculum. The TA muscle and tendon, as well as the retinaculum were undisturbed. Four to five tetani were performed with a 1-min rest interval to enable torque stabilization. The contribution of the tenotomized EDL muscle was negligible in this testing system.^13^ Peak TA muscle isometric torque was determined with the ankle at a right angle (0°), assuming a moment arm of 3 mm.^18^ *In vivo* isometric torque values were normalized to body weight.^18^

### Histology and immunohistology

TA muscles were embedded in a talcum-based gel, frozen in 2-methylbutane (Fisher Scientific), and super-cooled in liquid nitrogen using standard methodology previously reported.^17^ Frozen cross-sections (8 μm) were cut from the middle third of the TA muscle in the area where the original surgical defect was made. Sections were stained with hematoxylin and eosin (H&E), and oil red O, to assess tissue structure and cellularity, and fat deposition. Immunofluorescence was performed on sections probed for collagen I (1:500, AB755P; Millipore), sarcomeric myosin (MF20: 1:10; Hybridoma Bank), and nuclei (DAPI; 1:100; Invitrogen). Corresponding Alexaflour^®^ 488 and 596 labeled secondary antibodies (1:200–1:500; Invitrogen) were incubated at room temperature for 1 h. Qualitative assessments were made by observing three sections (separated by no less than 160 μm) from 3 to 5 muscles per group. In addition, the area fraction of collagen I, myosin, and oil-red-o in the defect area was quantified in nonoverlapping images using ImageJ (NIH), as previously reported.^13^

For assessment of muscles with GFP donor tissue, muscles were treated as described by Pilia et al.^19^ Cross- and longitudinal sections were probed for GFP (1:100) and myosin (MF20: 1:10; Hybridoma Bank) expression. Corresponding Alexaflour 488 and 596 labeled secondary antibodies (1:200–1:500; Invitrogen) were used for detection.

### Morphological assessment

Whole muscle H&E cross-sections were analyzed for total fiber number and muscle fiber morphological characteristics. Total fiber number was analyzed by manually counting each fiber in an entire TA muscle cross-section (*n*=3 muscles per group). Fiber morphological characteristics that included muscle fiber area, diameter (maximum Feret's diameter), and roundness were assessed in nonoverlapping images taken from the defect or remaining muscle mass regions of three TA muscles per group. Measurements were made by manually outlining each fiber using ImageJ (NIH), similar to methodology previously described.^17^ Only fibers with sizes between 50 and 7000 μm^2^ and a circularity between 0.3 and 1.0 were included for analysis to remove the measurement of neurovascular bundles and oblique fibers.^20^ These criteria excluded less than 1% of fibers analyzed in the defect region. Fibers on the border of the image were also excluded from analysis.

### Statistical analyses

Dependent variables were analyzed using one- and two-way analysis of variances (ANOVAs) and linear regression. Fisher's LSD *post-hoc* means comparisons testing was performed when a significant ANOVA was observed. The distribution frequencies of muscle fiber areas were compared using a Chi-Square test. Muscle fiber area frequencies were binned to 5 classifications (<1000, 1001–2000, 2001–3000, 3001–4000, and >4000 μm) requiring a *χ*^2^ of 9.49 to reach significance. Alpha was set at 0.05 for all tests. Values are listed as means±SEM (standard error of the mean). Statistical testing was performed using Microsoft Excel or Prism 6 for Mac (Graphpad).

## Results

### *Capacity of minced grafts to orchestrate* de novo *muscle fiber regeneration*

**FIG. 1.**
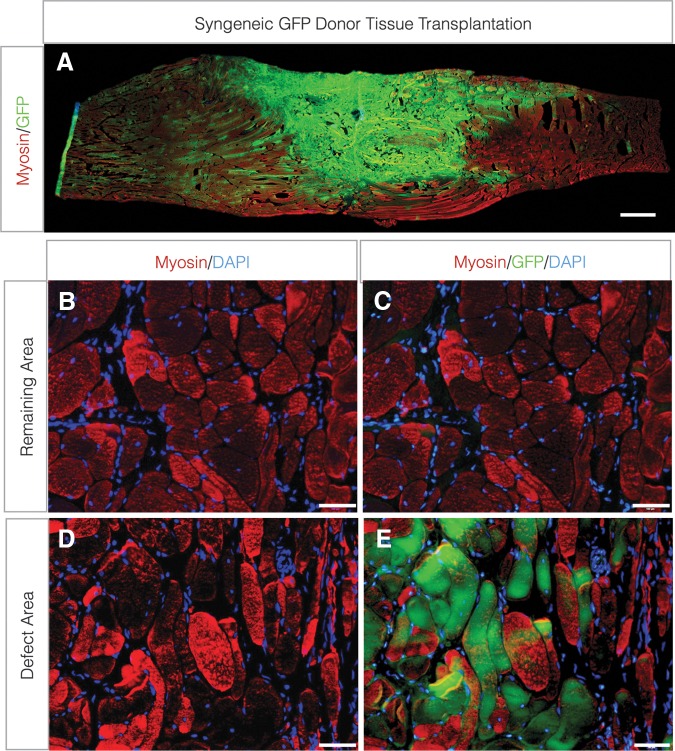
Contribution of donor-derived myogenic cells to *de novo* fiber regeneration after VML injury. TA muscle minced grafts derived from donor GFP-Lewis rats were transplanted to the site of VML injury at a 100% tissue replacement. **(A)** Longitudinal (scale bar=1.0 cm) and **(B–E)** cross-sections (scale bars=50 μm) from the remaining muscle mass **(B, C)** and defect area **(D, E)** were probed for sarcomeric myosin and GFP co-localization. TA, tibialis anterior; VML, volumetric muscle loss.

To determine whether minced graft-derived myogenic cells (i.e., primarily satellite cells delivered within their niche) contributed to *de novo* muscle fiber regeneration in a VML defect, grafts (100% replacement of excised tissue) were transplanted from syngeneic GFP donor rat TA muscles. Eight weeks postinjury, a large mass of GFP^+^ muscle fibers, which co-labeled for sarcomeric myosin, were observed (Fig. 1). The GFP^+^ fibers were localized primarily within the defect area, though some GFP^+^ fibers were observed proximal and distal to the defect area, suggesting migration of transplanted myogenic cells (Fig. 1B–E).

### TA muscle strength gains with minced graft volume expansion

**FIG. 2.**
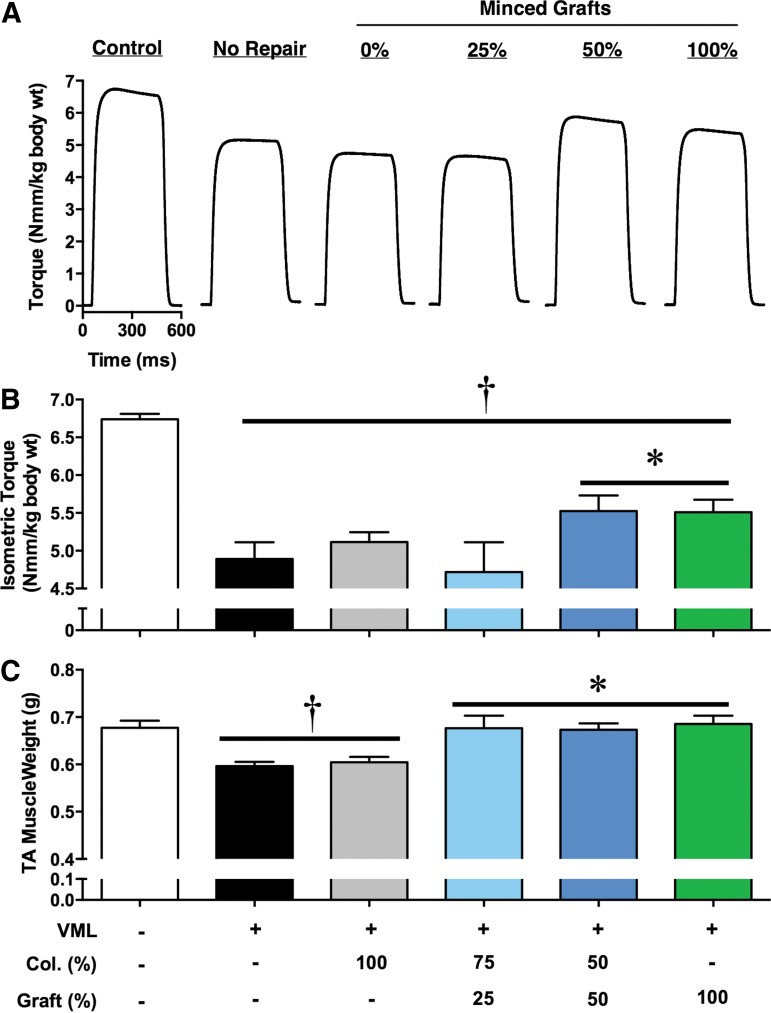
*In vivo* TA muscle torque production after treatment with autologous minced graft expansion constructs. Minced skeletal muscle grafts were transplanted to a TA muscle VML defect as a % of tissue defect volume. Grafts were delivered in a collagen hydrogel, reaching a similar volume (400 μL) per group. **(A)** Representative neural-evoked isometric tetanic torque waveforms (150 Hz; 400 msec train; 0.1 msec pulse width) and **(B)** group means±SEM are presented. **(C)** TA muscle wet weight. At the time of functional testing, body weights were similar among groups (414.3±3.3 g; ANOVA *p*=0.30). ^†^<Contralateral Control; *>all other VML injured groups, *p*<0.05. ANOVA, analysis of variance.

A potential limitation of the treatment of large clinical VML defects with autologous muscle is the availability of ample donor tissue and potential donor site complications and morbidity. Therefore, we sought to determine whether autologous minced muscle grafts could be volume expanded in a simple collagen hydrogel to achieve a similar functional recovery as a 100% minced graft replacement. To do so, *in vivo* neural-evoked torque of isolated TA muscles (no synergist muscle contribution) was measured (Fig. 2). In this model, VML injury without repair resulted in a 27% torque deficit 8 weeks postinjury compared with contralateral uninjured muscles. As a positive treatment control, a 100% minced graft tissue replacement restored 33% of the torque deficit. Volume expansion using 50% of the removed tissue achieved a similar (34%) restoration of the torque deficit; however, further graft volume expansion to 25% replacement of the tissue lost did not improve torque beyond VML-injured nonrepaired muscles. Repair of the VML defect only with collagen hydrogel (no minced grafts) also did not improve torque compared with nonrepaired muscles.

### Histological and immunohistological assessment of tissue regeneration

**FIG. 3.**
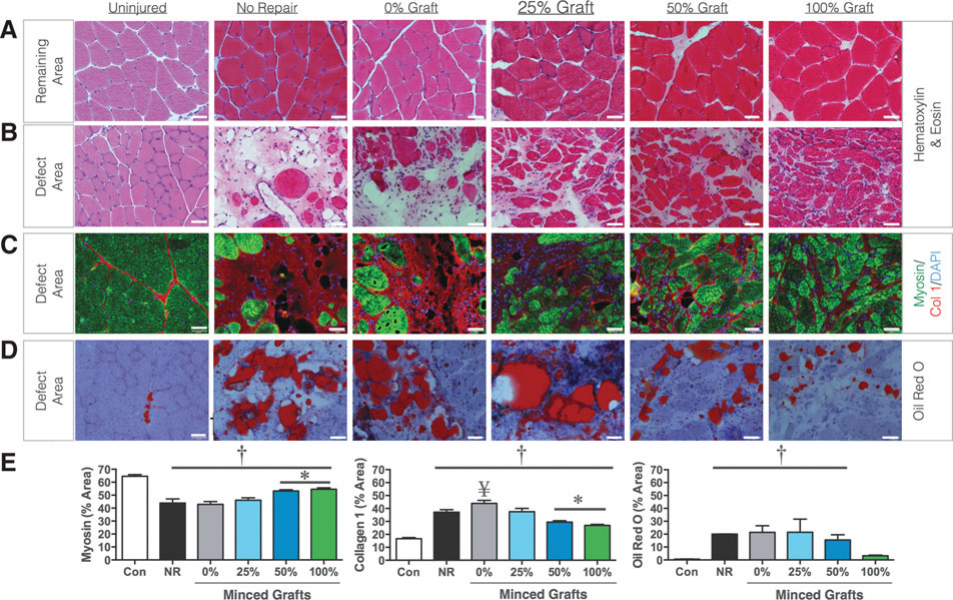
*De novo* muscle fiber regeneration within the defect area after treatment with autologous minced graft expansion constructs. Representative images from hematoxylin and eosin-stained cross-sections from the **(A)** remaining muscle mass area and **(B)** the defect area are presented. Cross-sections were probed for **(C)** myosin, collagen I and nuclei, and **(D)** oil red O. **(E)** Sections were semi-quantitated for area fraction of myosin, collagen I, and oil red O. Values are means±SEM. ^†^≠Contralateral Control; *≠all other VML injured groups; ^¥^>No Repair, *p*<0.05. All scale bars are 50 μm.

The remaining portion of the muscle after VML is not capable of endogenously regenerating the lost muscle tissue.^13,17,19,21^ This is reflected in TA muscle weights 8 weeks postinjury remaining 80 mg less than contralateral uninjured muscles, since the initial defect weight was ∼75 mg. However, TA muscle weights did not necessarily reflect functional recovery, since 25% graft repair presented fully recovered muscle weights despite no functional improvements (Fig. 2C). Therefore, histological analysis of the tissue was performed. VML-injured nonrepaired muscles presented few regenerated muscle fibers within the defect area, though the fibers in the remaining portion of the muscle appeared normal 8 weeks postinjury (Fig. 3A, B). Within the defect area, the density of muscle fibers appeared to increase with greater % volume graft repair (Fig. 3B). To confirm this observation, the area fraction of myosin and collagen was determined in images from the defect area (Fig. 3C). In comparison to contralateral uninjured muscles, all other groups exhibited lesser myosin and greater collagen I (Fig. 3E). However, the 50% and 100% graft repair promoted significantly greater myosin and lesser collagen I than all other experimental groups. In contrast, the presence of lipid deposition appeared to increase as the % volume graft repair decreased (Fig. 3D) repair with less than 100% graft repair resulting in greater lipid presence in the defect area (Fig. 3E).

### *Analysis of* de novo *muscle fiber regeneration*

**FIG. 4.**
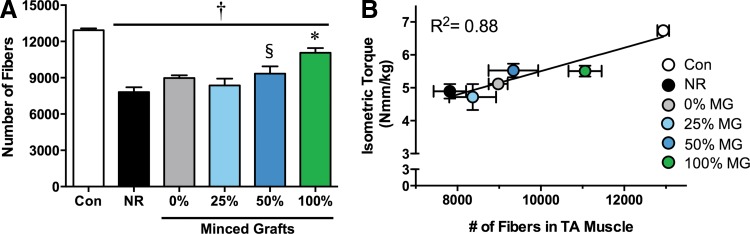
Quantification of *de novo* muscle fiber regeneration after treatment with autologous minced graft expansion constructs. **(A)** The number of muscle fibers per whole muscle cross-sectional area was manually counted. ^†^<Contralateral Control; *>all other VML injury groups ^§^>No Repair, *p*<0.05. **(B)** Regression analysis of fiber number to isometric torque per experimental group was performed. Values are means±SEM.

Strength gains after repair of VML are often attributed to *de novo* regeneration of muscle fibers throughout the literature, though this relationship has not been conclusively demonstrated.^22^ To assess *de novo* muscle fiber regeneration, the total number of muscle fibers in a cross-section from the middle third of the TA muscle was counted (Fig. 4A). Contralateral uninjured muscles comprised ∼13,000 muscle fibers, and the VML injury reduced the number of muscle fibers by ∼40% (∼7800 fibers in VML-injured nonrepaired muscles) 8 weeks postinjury. Though not significant, 0% and 25% graft volume repair resulted in a ∼15% and 7% increase in fiber number compared with nonrepaired muscles. Fifty percent graft repair significantly increased fiber number by ∼20% compared with no repair. One hundred percent graft volume repair resulted in a significant increase in fiber number compared with all other VML injured muscles (e.g., ∼41% >nonrepaired), but did not achieve full fiber restoration (i.e., ∼14% <contralateral uninjured muscle). Notably, regression analysis demonstrated a strong relationship between fiber number and isometric torque among groups (Fig. 4B: *R*^2^=0.88; *p*=0.005), indicating *de novo* regeneration of muscle fibers as a primary determinant of recovery of strength with increasing % graft volume repair.

### Morphological assessment of regenerated muscle fibers

**FIG. 5.**
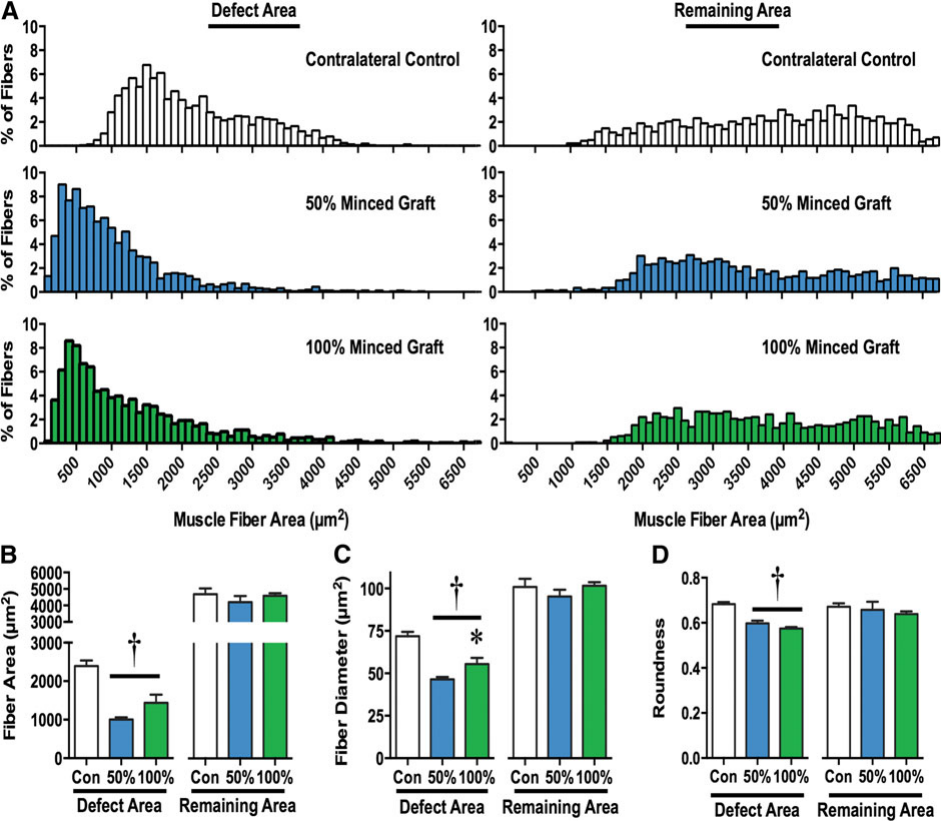
Morphological characterization of muscle fibers in VML injured muscles. Muscle fibers in the defect and remaining muscle mass area of uninjured contralateral, 50% minced graft-expanded, and 100% minced graft-repaired muscles were analyzed. **(A)** Muscle fiber area frequency distributions are presented. Muscle fiber **(B)** area, **(C)** diameter, **(D)** and roundness were quantified; values are means±SEM. ^†^<Contralateral Control; *>50% Minced Graft; *p*<0.05.

Although the 50% and 100% graft volume repair groups presented significantly greater fiber number, the morphology of regenerated fibers appeared less organized than uninjured tissue (Fig. 3). Therefore, muscle fiber morphological characteristics in the remaining and defect areas were analyzed (Fig. 5). In the remaining muscle area, there were no differences among contralateral uninjured, 50% and 100% graft groups for average fiber area, fiber diameter, or fiber roundness. In addition, the distribution of fiber area frequencies within the remaining muscle area of 50% and 100% graft groups was similar to uninjured contralateral control muscles (*χ*^2^=5.70 and 3.49, respectively). Within the defect area, both the 50% and 100% graft groups presented regenerated fibers with a smaller fiber area and diameter (Fig. 5B, C) and lesser roundness (Fig. 5D), suggesting an increased pennation angle. In addition, the distribution of fiber area frequencies within the defect area of 50% and 100% graft groups was significantly different from uninjured contralateral control muscles (*χ*^2^=796 and 544, respectively). Therefore, the fibers regenerated after graft transplantation are smaller and disoriented compared with uninjured muscles.

## Discussion

The most important finding of this study is that expanded autologous minced muscle grafts (50% less tissue) promote a similar functional recovery as a 100% graft repair. However, regenerative expansion was not fully supported in the collagen hydrogel, since only approximately half the number of fibers regenerated *de novo* with a 50% graft repair, compared with 100% repair. That being said, we conclusively demonstrate using GFP^+^ grafts that myogenic cells derived (e.g., satellite cells) from the minced grafts survive and directly contribute to significant *de novo* fiber regeneration within the defect area. Though the muscle tissue regenerated via minced grafts presented greater collagen content and altered histomorphometry compared with uninjured muscle, the fact that the 50% and 100% graft replacement promoted significant *de novo* muscle fiber regeneration supports the fact that an autologous muscle tissue-based approach may form a foundation on which restoration of functional muscle mass may be achieved after VML injury.

There was a corresponding decline in the number of fibers regenerated between 100% and 50% graft repair, and a precipitous decline from 50% to 25% graft repair, indicating that the collagen hydrogel did not provide ample support for expansion. One explanation is that the mechanical stability of the gel composition used (3 mg/mL collagen I) was not biomimetic of skeletal muscle.^23–25^ This possibility is in line with a recent paper, indicating that this formulation of collagen hydrogel supported ample vascularization after delivery of microvascular fragments to VML injured muscle, but did not support significant myogenesis.^19^ However, the biomaterial may not be the only limiting factor. Previously vital grafts co-delivered with devitalized muscle scaffolds (50% of each), which maintain many properties of muscle tissue,^26^ also did not appear to support graft expansion.^11^ Therefore, it is possible that *de novo* muscle tissue regeneration may improve with a myoconductive scaffold that not only provides a more informative matrix both structurally and compositionally (e.g., inclusion of basal lamina) but also may require adjunct therapies such as alternative myogenic cells,^27^ growth factors/small molecules,^28^ and/or physical rehabilitation.^13^

Autologous minced graft expansion and ECM-mediated regeneration may be primarily restricted by a similar mechanism, as both rely on satellite cell migration for *de novo* fiber regeneration. Previously, we and other labs have demonstrated that ECMs derived from muscle and other tissues (e.g., urinary bladder matrix & small intestine submucosa) do not promote appreciable muscle fiber regeneration beyond ∼0.5 mm from the remaining muscle mass, if at all.^17,22,29–33^ The collagen hydrogel used in this study was also ineffective in promoting muscle fiber regeneration. Collectively, these findings indicate that providing a matrix alone is not sufficient to promote appreciable muscle fiber regeneration in a VML defect. In a recent investigation, the restricted *de novo* muscle fiber regeneration mediated by ECMs was partly attributed to an impaired migration of satellite cells (Pax7^+^) from the remaining portion of the host muscle to the scaffolding in the defect area.^26^ The findings of this study that (1) minced graft-derived myogenic cells directly mediate *de novo* fiber regeneration, (2) *de novo* fiber regeneration is directly related to the amount of minced graft replacement (i.e, myogenic cells), and (3) collagen–hydrogel alone does not support fiber regeneration, as well as previous reports that devitalized scaffolds (which maintain many properties of muscle tissue)^26^ and small muscle grafts devoid of functional satellite cells (Pax7 knockout)^34^ do not promote regeneration, highlight that inclusion of myogenic cells (satellite cells) is necessary for *de novo* fiber regeneration. Significant intramuscular satellite cell migration has been observed in mice with focal muscle injury^35^ and, to that end, we observed putative satellite cell migration from minced grafts to the remaining muscle mass in this study, which indicates that the inherent ability of satellite cells to migrate is not the limiting factor *per se*. Instead, the pro-fibrotic environment created by VML^2,36^ likely restricts satellite cell migration during graft expansion and ECM-mediated regeneration, and, therefore, further exploration of approaches to improve myogenic cell migration by attenuating ensuing inflammatory and fibrotic responses is warranted.

A strong correlation existed among muscle fiber number and strength among VML injured and repaired groups, indicating that *de novo* muscle fiber regeneration is a primary determinant of strength recovery. However, inspection of this relationship between 100% and 50% minced graft groups indicates multiple mechanisms of functional recovery. VML studies conducting functional assessments have reported strength gains related to either appreciable *de novo* fiber regeneration within the defect (increased force production)^13^ or ECM deposition within the defect that improves transmission of force produced by the remaining muscle mass.^17^ Given that the 50% graft group regenerated only approximately half of the muscle fibers in the 100% graft group, it is possible that the 50% graft group may be evidence of both mechanisms, that is, improved force production and transmission. However, significant strength improvements in this study were not observed in groups that did not present significant *de novo* fiber regeneration but presented collagen deposition in the defect (e.g., 25% graft), suggesting that other unidentified mechanisms may underlie the equivalent strength improvements with 50% graft repair.

Minced graft transplantation (50% and 100%) promoted significant *de novo* fiber regeneration; however, the regenerated tissue composition and fiber morphology was altered compared with uninjured muscle. Notably, the regenerated tissue presented greater collagen and lipid deposition (50% but not 100%) and the fibers had a smaller average cross-sectional area than in uninjured muscle. These characteristics likely contributed to a lesser than expected functional recovery estimated from the number of fibers regenerated. In addition, the roundness of the fibers was reduced, suggesting that the regenerated fibers were pennated more than in uninjured muscle. It is unclear in this study whether this is a positive or negative adaptation. While an increased pennation angle reduces the contribution of a fiber's force to net muscle torque, increasing pennation angle also allows for more muscle fibers to employ a given area and, therefore, may ultimately result in an increased net torque production.^37^ These findings and those previously made of altered muscle length-tension properties after VML^2^ highlight the influence of muscle architecture in the pathophysiology and repair of VML.

Despite the altered histomorphology, it is important to note that the muscle tissue regeneration after VML repair using 50% or 100% graft repair appears to be greater than with decellularized ECM-based approaches.^17,22,31–33,38–41^ Moreover, the re-establishment of a portion of the lost muscle tissue provides the opportunity for further muscle tissue augmentation using physical rehabilitation to improve regenerative and physiological outcomes that would not otherwise be possible.

In addition to efficacy and safety, donor tissue availability is recognized as a potential limitation to clinical translation of minced grafts for the repair of large VML defects in large muscles (e.g., human quadriceps; ∼1 L^42^), but not necessarily in smaller muscles (e.g., human TA; 0.15 L^43^). For example, the quadriceps with a 70% VML would require 0.7 (740), 0.35 (370), and 0.18 (190) L (or grams with muscle density of 1.056 g/mL) of donor tissue for a 100%, 50%, and 25% tissue replacement; the TA muscle with a 70% VML would require 0.11 (116), 0.053 (56), and 0.026 (27) L (grams) of donor tissue, respectively. The findings of this study indicate that a standard latissimus dorsi flap, which provides ∼0.26 L (∼275 grams; assuming 1 cm thickness) of muscle tissue,^44,45^ approaches enough tissue to support minced graft expansion for repair of large muscles and fully supports applications in smaller muscles.

The ultimate goal of this line of research is to regenerate muscle tissue and restore functional capacity after clinical VML. Since VML is heterogenous among patients,^2,16,22^ clinicians would benefit from a variety of surgical and regenerative treatment options. Xenogeneic decellularized extracellular matrices, as have recently been tested in the clinic,^16,22^ embody an ideal treatment for VML in that no autologous donor tissue is required. Transplantation of ECMs to VML defects in six patients has promoted modest gains in strength with no reported adverse outcomes.^16,22^ However, the histological data reported from different laboratories using a variety of ECMs in various VML animal models do not support ECMs role in promoting appreciable muscle fiber regeneration.^17,22,30–33,46^ On the other hand, minced grafts directly contribute to and orchestrate appreciable *de novo* muscle regeneration,^10–13,26,47,48^ but the amount of autologous donor muscle tissue required to repair large VML may be limited. To address this potential limitation, the capacity of minced grafts for volume expansion in collagen was tested. The results of the study did not support graft expansion in this biomaterial, but indicated that that use of 50% less tissue promoted similar functional improvements as 100% graft repair. Among other barriers to regenerate large volumes of skeletal muscle such as revascularization (e.g., Pilia et al.^19^), restricted satellite cell migration is likely a significant barrier to minced graft expansion and ECM-mediated regeneration.

## Abbreviations Used

ANOVA: analysis of variance
ECM: extracellular matrix
EDL: extensor digitorum longus
TA: tibialis anterior
VML: volumetric muscle loss

## References

[1] Doukas WC, Hayda RA, Frisch HM, et al. The Military Extremity Trauma Amputation/Limb Salvage (METALS) study: outcomes of amputation versus limb salvage following major lower-extremity trauma. J Bone Joint Surg Am. 2013;95:138–145 23324961 10.2106/JBJS.K.00734

[2] Garg K, Ward CL, Hurtgen BJ, et al. Volumetric muscle loss: persistent functional deficits beyond frank loss of tissue. J Orthop Res. 2015;33:40–46 25231205 10.1002/jor.22730

[3] Burns TC, Stinner DJ, Possley DR, et al. Does the zone of injury in combat-related Type III open tibia fractures preclude the use of local soft tissue coverage? J Orthop Trauma. 2010;24:697–703 20926962 10.1097/BOT.0b013e3181d048b8

[4] Lin CH, Lin YT, Yeh JT, et al. Free functioning muscle transfer for lower extremity posttraumatic composite structure and functional defect. Plast Reconstr Surg. 2007;119:2118–2126 17519710 10.1097/01.prs.0000260595.85557.41

[5] Faulkner JA, Carlson BM, Kadhiresan VA Review: whole skeletal muscle transplantation: mechanisms responsible for functional deficits. Biotechnol Bioeng. 1994;43:757–763 18615799 10.1002/bit.260430810

[6] Myeroff C, Archdeacon M Autogenous bone graft: donor sites and techniques. J Bone Joint Surg Am. 2011;93:2227–2236 22159859 10.2106/JBJS.J.01513

[7] Epstein NE A preliminary study of the efficacy of Beta Tricalcium Phosphate as a bone expander for instrumented posterolateral lumbar fusions. J Spinal Disord Tech. 2006;19:424–429 16891978 10.1097/00024720-200608000-00009

[8] Epstein NE Efficacy of different bone volume expanders for augmenting lumbar fusions. Surg Neurol. 2008;69:16–19; discussion 9 18054607 10.1016/j.surneu.2007.03.021

[9] Carlson BM Regeneration fo the completely excised gastrocnemius muscle in the frog and rat from minced muscle fragments. J Morphol. 1968;125:447–472 5687378 10.1002/jmor.1051250405

[10] Carlson BM, Gutmann E Development of contractile properties of minced muscle regenerates in the rat. Exp Neurol. 1972;36:239–249 5053353 10.1016/0014-4886(72)90020-9

[11] Ghins E, Colson-Van Schoor M, Marechal G The origin of muscle stem cells in rat triceps surae regenerating after mincing. J Muscle Res Cell Motil. 1984;5:711–722 6533161 10.1007/BF00713929

[12] Snow MH Metabolic activity during the degenerative and early regenerative stages of minced skeletal muscle. Anat Rec. 1973;176:185–203 4351200 10.1002/ar.1091760207

[13] Corona BT, Garg K, Ward CL, et al. Autologous minced muscle grafts: a tissue engineering therapy for the volumetric loss of skeletal muscle. Am J Physiol Cell Physiol. 2013;305:C761–C175 23885064 10.1152/ajpcell.00189.2013

[14] Gras S, Klarskov N, Lose G Intraurethral injection of autologous minced skeletal muscle: a simple surgical treatment for stress urinary incontinence. J Urol. 2014;192:850–855 24735937 10.1016/j.juro.2014.04.005

[15] Schneider U, Rackwitz L, Andereya S, et al. A prospective multicenter study on the outcome of type I collagen hydrogel-based autologous chondrocyte implantation (CaReS) for the repair of articular cartilage defects in the knee. Am J Sports Med. 2011;39:2558–2565 21984690 10.1177/0363546511423369

[16] Mase VJ Jr., Hsu JR, Wolf SE, et al. Clinical application of an acellular biologic scaffold for surgical repair of a large, traumatic quadriceps femoris muscle defect. Orthopedics. 2010;33:511 20608620 10.3928/01477447-20100526-24

[17] Corona BT, Wu X, Ward CL, et al. The promotion of a functional fibrosis in skeletal muscle with volumetric muscle loss injury following the transplantation of muscle-ECM. Biomaterials. 2013;34:3324–3335 23384793 10.1016/j.biomaterials.2013.01.061

[18] Johnson WL, Jindrich DL, Roy RR, et al. A three-dimensional model of the rat hindlimb: musculoskeletal geometry and muscle moment arms. J Biomech. 2008;41:610–619 18061600 10.1016/j.jbiomech.2007.10.004 PMC2322854

[19] Pilia M, Mcdaniel JS, Guda T, et al. Transplantation and perfusion of microvascular fragments in a rodent model of volumetric muscle loss injury. Eur Cell Mater. 2014;28:11–24 25017641 10.22203/ecm.v028a02

[20] Meyer GA, Lieber RL Skeletal muscle fibrosis develops in response to desmin deletion. Am J Physiol Cell Physiol. 2012;302:C1609–C1620 22442138 10.1152/ajpcell.00441.2011 PMC3378016

[21] Machingal MA, Corona BT, Walters TJ, et al. A tissue-engineered muscle repair construct for functional restoration of an irrecoverable muscle injury in a murine model. Tissue Eng Part A. 2011;17:2291–2303 21548710 10.1089/ten.tea.2010.0682 PMC3161107

[22] Sicari BM, Rubin JP, Dearth CL, et al. An acellular biologic scaffold promotes skeletal muscle formation in mice and humans with volumetric muscle loss. Sci Transl Med. 2014;6:234–258 10.1126/scitranslmed.3008085 PMC5942588 24786326

[23] Feng CH, Cheng YC, Chao PH The influence and interactions of substrate thickness, organization and dimensionality on cell morphology and migration. Acta Biomater. 2013;9:5502–5510 23201017 10.1016/j.actbio.2012.11.024

[24] Mori H, Lo AT, Inman JL, et al. Transmembrane/cytoplasmic, rather than catalytic, domains of Mmp14 signal to MAPK activation and mammary branching morphogenesis via binding to integrin beta1. Development. 2013;140:343–352 23250208 10.1242/dev.084236 PMC3597211

[25] Lv S, Dudek DM, Cao Y, et al. Designed biomaterials to mimic the mechanical properties of muscles. Nature. 2010;465:69–73 20445626 10.1038/nature09024

[26] Garg K, Ward CL, Rathbone CR, et al. Transplantation of devitalized muscle scaffolds is insufficient for appreciable *de novo* muscle fiber regeneration after volumetric muscle loss injury. Cell Tissue Res 2014;358:857–873 25300647 10.1007/s00441-014-2006-6

[27] Camargo FD, Green R, Capetanaki Y, et al. Single hematopoietic stem cells generate skeletal muscle through myeloid intermediates. Nat Med. 2003;9:1520–1527 14625546 10.1038/nm963

[28] Sacco A, Doyonnas R, Labarge MA, et al. IGF-I increases bone marrow contribution to adult skeletal muscle and enhances the fusion of myelomonocytic precursors. J Cell Biol. 2005;171:483–492 16275752 10.1083/jcb.200506123 PMC2171272

[29] Brown BN, Valentin JE, Stewart-Akers AM, et al. Macrophage phenotype and remodeling outcomes in response to biologic scaffolds with and without a cellular component. Biomaterials. 2009;30:1482–1491 19121538 10.1016/j.biomaterials.2008.11.040 PMC2805023

[30] Ma J, Sahoo S, Baker AR, et al. Investigating muscle regeneration with a dermis/small intestinal submucosa scaffold in a rat full-thickness abdominal wall defect model. J Biomed Mater Res B Appl Biomater 2015;103:355–364 24889422 10.1002/jbm.b.33166

[31] Merritt EK, Hammers DW, Tierney M, et al. Functional assessment of skeletal muscle regeneration utilizing homologous extracellular matrix as scaffolding. Tissue Eng Part A. 2010;16:1395–1405 19929169 10.1089/ten.TEA.2009.0226

[32] Sicari BM, Agrawal V, Siu BF, et al. A murine model of volumetric muscle loss and a regenerative medicine approach for tissue replacement. Tissue Eng Part A. 2012;18:1941–1948 22906411 10.1089/ten.tea.2012.0475 PMC3463275

[33] Turner NJ, Badylak JS, Weber DJ, et al. Biologic scaffold remodeling in a dog model of complex musculoskeletal injury. J Surg Res. 2012;176:490–502 22341350 10.1016/j.jss.2011.11.1029

[34] Sambasivan R, Yao R, Kissenpfennig A, et al. Pax7-expressing satellite cells are indispensable for adult skeletal muscle regeneration. Development. 2011;138:3647–3656 21828093 10.1242/dev.067587

[35] Schultz E, Jaryszak DL, Valliere CR Response of satellite cells to focal skeletal muscle injury. Muscle Nerve. 1985;8:217–222 4058466 10.1002/mus.880080307

[36] Garg K, Corona BT, Walters TJ Losartan administration reduces fibrosis but hinders functional recovery after volumetric muscle loss injury. J Appl Physiol (1985). 2014;117:1120–1131 25257876 10.1152/japplphysiol.00689.2014

[37] Lieber RL, Friden J Functional and clinical significance of skeletal muscle architecture. Muscle Nerve. 2000;23:1647–1666 11054744 10.1002/1097-4598(200011)23:11<1647::aid-mus1>3.0.co;2-m

[38] Chen XK, Walters TJ Muscle-derived decellularised extracellular matrix improves functional recovery in a rat latissimus dorsi muscle defect model. J Plast Reconstr Aesthet Surg 2013;66:1750–1758 24007646 10.1016/j.bjps.2013.07.037

[39] Corona BT, Ward CL, Baker HB, et al. Implantation of *in vitro* tissue engineered muscle repair constructs and bladder acellular matrices partially restore *in vivo* skeletal muscle function in a rat model of volumetric muscle loss injury. Tissue Eng Part A. 2014;20:705–715 24066899 10.1089/ten.tea.2012.0761 PMC4518882

[40] Gamba PG, Conconi MT, Lo Piccolo R, et al. Experimental abdominal wall defect repaired with acellular matrix. Pediatr Surg Int. 2002;18:327–331 12415348 10.1007/s00383-002-0849-5

[41] Merritt EK, Cannon MV, Hammers DW, et al. Repair of traumatic skeletal muscle injury with bone-marrow-derived mesenchymal stem cells seeded on extracellular matrix. Tissue Eng Part A. 2010;16:2871–2881 20412030 10.1089/ten.TEA.2009.0826

[42] Trappe TA, Lindquist DM, Carrithers JA Muscle-specific atrophy of the quadriceps femoris with aging. J Appl Physiol (1985). 2001;90:2070–2074 11356767 10.1152/jappl.2001.90.6.2070

[43] Fukunaga T, Roy RR, Shellock FG, et al. Physiological cross-sectional area of human leg muscles based on magnetic resonance imaging. J Orthop Res. 1992;10:928–934 1403308 10.1002/jor.1100100623

[44] Bailey BN, Godfrey AM Latissimus dorsi muscle free flaps. Br J Plast Surg. 1982;35:47–52 7039752 10.1016/0007-1226(82)90083-2

[45] Kim SW, Jeon SB, Hwang KT, et al. Coverage of amputation stumps using a latissimus dorsi flap with a serratus anterior muscle flap: a comparative study. Ann Plast Surg. 2014 [Epub ahead of print]; DOI: 10.1097/SAP.0000000000000220 25003415

[46] Brown BN, Londono R, Tottey S, et al. Macrophage phenotype as a predictor of constructive remodeling following the implantation of biologically derived surgical mesh materials. Acta Biomater. 2012;8:978–987 22166681 10.1016/j.actbio.2011.11.031 PMC4325370

[47] Carlson BM The Regeneration of Minced Muscles. S. Karger: Basel, NY, 1972. 4577782

[48] Ghins E, Colson-Van Schoor M, Marechal G Implantation of autologous cells in minced and devitalized rat skeletal muscles. J Muscle Res Cell Motil. 1986;7:151–159 3711311 10.1007/BF01753416

